# Diagnostic Performance and Interobserver Agreement of the Vesical Imaging–Reporting and Data System (VI-RADS) in Bladder Cancer Staging: A Systematic Review

**DOI:** 10.3390/medicina61030469

**Published:** 2025-03-07

**Authors:** Alexandru Nesiu, Dorin Novacescu, Silviu Latcu, Razvan Bardan, Alin Cumpanas, Flavia Zara, Victor Buciu, Radu Caprariu, Talida Georgiana Cut, Ademir Horia Stana

**Affiliations:** 1 Discipline of Urology, Department of Medicine, Vasile Goldiş Western University, Liviu Rebreanu Boulevard, No. 86, 310414 Arad, Romania; nesiu.alexandru@uvvg.ro; 2 Department II of Microscopic Morphology, “Victor Babes” University of Medicine and Pharmacy Timisoara, E. Murgu Square, No. 2, 300041 Timisoara, Romania; flavia.zara@umft.ro; 3 Doctoral School, “Victor Babes” University of Medicine and Pharmacy Timisoara, E. Murgu Square, No. 2, 300041 Timisoara, Romania; silviu.latcu@umft.ro (S.L.); victor.buciu@umft.ro (V.B.); 4 Discipline of Urology, Department XV, “Victor Babes” University of Medicine and Pharmacy Timisoara, E. Murgu Square, No. 2, 300041 Timisoara, Romania; razvan.bardan@umft.ro (R.B.); cumpanas.alin@umft.ro (A.C.); 5 Discipline of Radiology and Medical Imaging, Department XV, “Victor Babes” University of Medicine and Pharmacy Timisoara, E. Murgu Square, Nr. 2, 300041 Timisoara, Romania; radu.caprariu@umft.ro; 6 Discipline of Infectious Diseases, Department XIII, “Victor Babes” University of Medicine and Pharmacy Timisoara, E. Murgu Square, No. 2, 300041 Timisoara, Romania; talida.cut@umft.ro; 7 Discipline of Radiology, Department of Medicine, Vasile Goldiş Western University, Liviu Rebreanu Boulevard, No. 86, 310414 Arad, Romania; stana.ademir-horia@uvvg.ro

**Keywords:** vesical imaging–reporting and data system (VI-RADS), bladder cancer diagnosis and staging, urothelial cancer, multiparametric magnetic resonance imaging (mp-MRI), non-muscle-invasive bladder cancer (NMIBC), muscle-invasive bladder cancer (MIBC), interobserver agreement, systematic review, diagnostic accuracy, urologic oncology imaging

## Abstract

*Background and Objectives*: The Vesical Imaging–Reporting and Data System (VI-RADS) represents a standardized approach for interpreting multiparametric magnetic resonance imaging (mp-MRI) in bladder cancer (BC) evaluation. This systematic review aimed to assess the VI-RADS’ diagnostic performance and interobserver agreement in distinguishing muscle-invasive from non-muscle-invasive BC, a crucial differentiation for treatment planning. *Materials and Methods*: A systematic literature search was conducted through PubMed, Google Scholar, and Web of Science, over an initial five-year time span, from VI-RADS’ inception (May 2018) to November 2023. Studies reporting VI-RADS’ diagnostic performance with histopathological confirmation and interobserver agreement data were included. The diagnostic accuracy was assessed using sensitivity and specificity, while interobserver agreement was evaluated using Cohen’s κ coefficient. *Results*: Nine studies comprising 1249 participants met the inclusion criteria. Using a VI-RADS score cutoff of ≥3, the pooled sensitivity and specificity for detecting muscle invasion were 88.2% and 80.6%, respectively. Interobserver agreement showed excellent consistency with a mean κ value of 0.82. Individual study sensitivities ranged from 74.1% to 94.6%, while specificities varied from 43.9% to 96.5%. *Conclusions*: VI-RADS demonstrates high diagnostic accuracy and excellent interobserver agreement in BC staging, supporting its role as a reliable non-invasive diagnostic tool. However, it should be used as a complementary tool to, not a replacement for, histopathological confirmation. Moreover, the variability in specificity suggests the need for standardized training and interpretation protocols. Clinical correlation and adequate reader experience are essential for optimal implementation. Future integration with pathological data may further enhance its predictive value.

## 1. Introduction

Bladder cancer (BC) is one of the most frequently encountered urological pathologies—especially amongst men—being the 11th most common cancer in adults globally [1]. The incidence of this disease is highly skewed towards men—9.5, compared to 2.4 for females [2]. Smoking is, by far, the most incriminated risk factor for BC [3], although the risk has not yet been associated with electronic cigarettes. On the other hand, neither a family history of BC nor metabolic risk factors, such as dyslipidemia or hypertension, have been shown to be associated with the present risk of development [4,5].

The most prevalent subtype of BC is urothelial (i.e., transitional cell) carcinoma, which makes up 85–90% of all cases. The most important differentiating criterion in the treatment of BC is the invasion of the detrusor muscle. As such, we differentiate BC based on the staging of the lesion in relation to the detrusor muscle—into invasive (≥T2 stage) and non-muscle-invasive (≤T1 stage).

While non-muscle-invasive BC (NMIBC) is primarily managed with transurethral resection of the bladder tumor (TUR-BT), accompanied by intravesical chemotherapeutic adjuvant instillation therapy, the management of muscle-invasive BC (MIBC) is based on radical cystectomy (RC) with external radiotherapy, systemic chemotherapy, or a combination of the two [6,7]. Given those different treatment plans, the aforementioned distinction is highly important to make correctly.

The most common symptom of NMIBC is macroscopic hematuria, which is associated with a more advanced stage than its microscopic counterpart. The diagnosis relies mainly on ultrasonography, cystoscopy, computer tomography (CT), or magnetic resonance imaging (MRI).

Due to recent advances, multiparametric MRI (mp-MRI), including T2-weighted images, diffusion-weighted images, and dynamic contrast-enhanced images, is currently considered a reasonable means to facilitate decision making in BC [8,9,10]. The Vesical Imaging–Reporting and Data System (VI-RADS) was released almost five years prior to this paper being written, in May 2018, and was meant to be the standard imaging method for the diagnosis of BC [11].

One of the most seemingly prevalent limitations to this method of diagnosis is the need for an experienced mp-MRI radiologist. Thus, some studies have compared the results from both radiologists and clinicians with different skill levels to identify how results may vary. To best underline potential weaknesses and to best inform future updates of VI-RADS, this study aimed to review and evaluate the interobserver agreement and diagnostic performance of VI-RADS for BC.

## 2. Materials and Methods

Prior to conducting this systematic review, we developed a detailed protocol specifying the search strategy, eligibility criteria, data extraction methods, and planned analyses. While the protocol was not published, it was registered in PROSPERO (CRD42025642149). Any deviations from the original protocol are noted in the study limitations section.

We systematically performed the literature search through PubMed, Google Scholar, and Web of Science for eligible studies from inception up to November 2023. The search strategy combined Medical Subject Headings (MeSH) terms and free-text keywords related to BC imaging and VI-RADS. The primary search string included (“VI-RADS” OR “vesical imaging reporting and data system”) AND (“bladder cancer” OR “bladder carcinoma” OR “bladder tumor”) AND (“magnetic resonance imaging” OR “MRI” OR “multiparametric MRI”). Additional relevant articles were identified through manual screening of reference lists from the selected studies and review articles. The current systematic review was carried out in accordance with the latest recommendations of the Preferred Reporting Items for Systematic and Meta-analyses (PRISMA) [12].

Study selection followed a two-stage process. First, we screened titles and abstracts for potential eligibility. Subsequently, potentially eligible articles were evaluated against predefined inclusion criteria: (1) studies that aimed to report the performance of VI-RADS in detecting either NMIBC, MIBC, or both and assess interobserver variability; (2) the presence of muscle invasion, and the malignant nature of the tumor was postoperatively confirmed through histological exam; (3) studies published in a peer-reviewed English-language journal, with the full text available for quality assessment.

Exclusion criteria were set as follows: (1) replies, comments, case reports, letters, editorials, or conference abstracts; (2) studies lacking histopathological correlation; (3) studies with inadequate information for data extraction or quality assessment, focused solely on technical aspects without diagnostic performance data; (4) non-English papers; (5) studies presenting overlapping patient populations with other included studies.

The data for statistical analysis were pooled and compiled using Microsoft Excel 2016 and Microsoft Word 2016. The level of confidence was set at 95%, resulting in a *p*-value < 0.05 to indicate a significant association. The degree of interobserver agreement was quantified by Cohen’s κ statistic (K). The K value can be interpreted as interobserver agreement being poor (<0.20), fair (0.21–0.40), moderate (0.41–0.60), good (0.61–0.80), and excellent (0.81–1.00). We mention that some of the included studies had more than two observers, thus resulting in more than one K value. When centralizing the data, the lowest value for the K coefficient was selected. This, in turn, considers the worst possible outcome of one given paper, thus making the final result of this study more reliable and reproducible in a heterogeneous population.

Statistical analysis was performed using Comprehensive Meta-Analysis Software Version 3.0 (Biostat, Englewood, NJ, USA) and Review Manager (RevMan) Version 5.4 (The Nordic Cochrane Centre, Copenhagen, Denmark). The meta-analysis was conducted following the PRISMA guidelines. For each included study, histopathological confirmation through either RC specimens or TUR-BT (with or without a second look) served as the reference standard. Assessment of heterogeneity was performed using the I^2^ statistic, with values > 75% indicating substantial heterogeneity. Due to the significant heterogeneity observed among studies (I^2^ = 82%), we determined that a formal meta-analysis would not provide meaningful pooled estimates. Therefore, we conducted a systematic review with a narrative synthesis of the findings.

**Figure 1:**
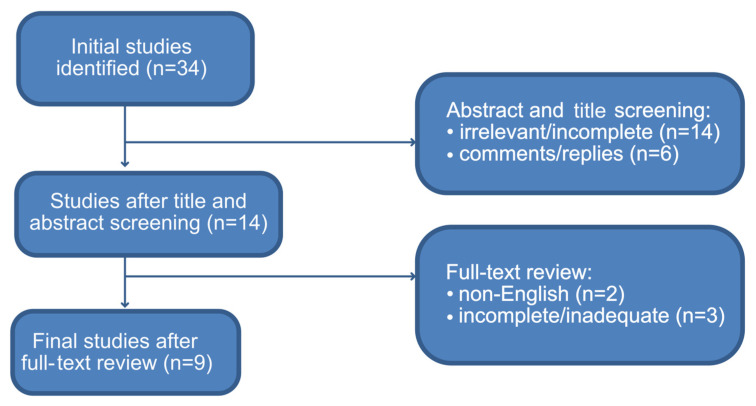
Flow diagram of search algorithm.

In total, 34 articles were retrieved by the literature search. The title and abstract were screened—14 articles were excluded for irrelevant content or incomplete information and 6 for being comments and replies. After that, the full text was reviewed and two studies were excluded for non-English language use and another three studies for further inadequate or incomplete methodological reporting, lacking parts of the required information to be retrieved. Finally, nine articles [13,14,15,16,17,18,19,20,21] including interobserver variability in VI-RADS score assessment were included (see Figure 1).

The methodological quality and risk of bias of the included studies were assessed using the Quality Assessment of Diagnostic Accuracy Studies-2 (QUADAS-2) tool. Two independent reviewers (A.N. and D.N.) evaluated each study across four key domains: patient selection, index test (VI-RADS), reference standard (histopathology), and study flow and timing. For each domain, the risk of bias was classified as low, high, or unclear. The reviewers assessed the applicability of the first three domains to the review question. Disagreements were resolved through consensus, with a third reviewer (A.H.S.) consulted when necessary. Publication bias was evaluated through visual inspection of funnel plots and Deeks’ test for funnel plot asymmetry.

To explore potential sources of heterogeneity in diagnostic accuracy estimates, we conducted predetermined subgroup analyses. Studies were stratified based on MRI field strength (1.5 T versus 3.0 T), study size (more or less than 100 participants), and reader experience with VI-RADS (more or less than 5 years). Meta-regression analyses examined the relationship between these study characteristics and diagnostic performance measures. Sensitivity analyses assessed the robustness of our findings through three approaches: excluding studies with a high risk of bias in any QUADAS-2 domain, comparing different VI-RADS thresholds (≥4 versus ≥3), and separate analysis of single-center versus multi-center studies. These analyses helped us evaluate the stability of our pooled estimates under different methodological assumptions.

Differentiating NMIBC and MIBC is crucial for counseling patients with BC. Surgical management of NMIBC and MIBC is completely different and using mp-MRI to differentiate it might reduce costs, perioperative complications, morbidity, and mortality. Different results were obtained considering different VI-RADS cutoffs. For this study, we considered the cutoff point between NMIBC and MIBC to be VI-RADS 3 (≥3). The sensitivity and specificity were pooled from each study, together with the area under the receiver operating characteristic curve (AUROC) for the effectiveness of the diagnosis model, Cohen’s kappa coefficient (K) for interobserver agreement, and the number of patients included in each study.

The overall certainty of evidence was evaluated using the Grading of Recommendations Assessment, Development and Evaluation (GRADE) approach modified for diagnostic accuracy studies. For each outcome, we assessed five domains: risk of bias (using QUADAS-2 results), inconsistency (heterogeneity in diagnostic accuracy estimates), indirectness (applicability to the review question), imprecision (width of confidence intervals and sample size), and publication bias. The certainty of evidence was categorized as high (further research unlikely to change our confidence in results), moderate (further research may impact our confidence), low (further research likely to impact our confidence), or very low (any estimate of effect is uncertain).

## 3. Results

### 3.1. Definitions, Procedural Details, and Clinical Considerations

Although apparently beyond the scope of the clinician, a deeper understanding of the mp-MRI and the VI-RADS scoring system should prove to be invaluable in the face of this rapidly growing, and so far under-regulated, diagnostic approach. In this section, our paper will shortly go over radiological considerations of VI-RADS, underlining specific key points that are required for adequate mp-MRI reading and clinical associations.

For the purpose of this study, we describe, in short, the clinical implications of the VI-RADS scoring system in Table 1.

**Figure 2:**
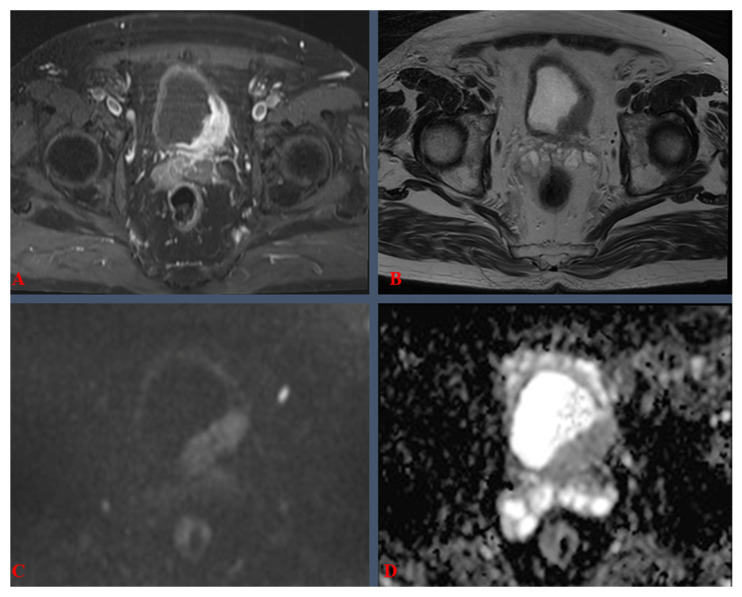
Multiparametric magnetic resonance imaging of the pelvis, at initial presentation of a bladder tumor case, scored as VI-RADS 4, on a 1.5-Tesla machine: (**A**) post-contrast fat-saturated axial T1-weighted sequence; (**B**) axial T2-weighted sequence; (**C**) diffusion-weighted imaging (DWI), axial sequence; (**D**) apparent diffusion coefficient (ADC) axial map. The mean ADC value for this tumor was 0.85 × 10^−3^ mm^2^/s.

**Figure 3:**
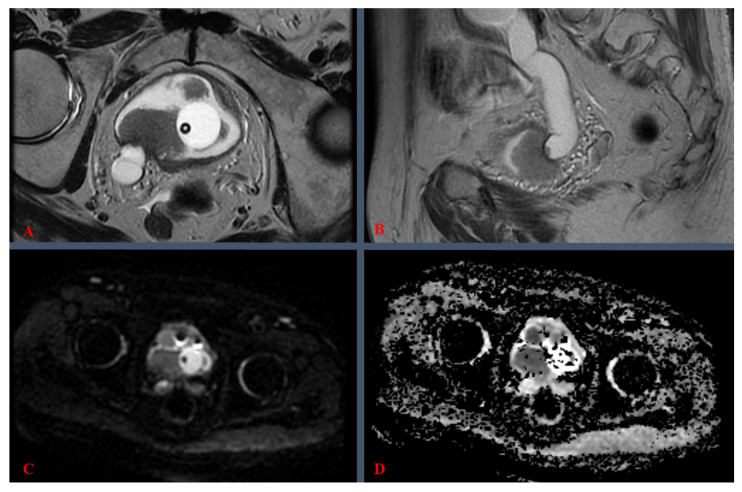
Multiparametric magnetic resonance imaging of the pelvis, at initial presentation of a bladder tumor case, with an indwelling Foley catheter, scored as VI-RADS 5, on a 1.5-Tesla machine: (**A**) axial T2-weighted sequence; (**B**) sagittal T2-weighted sequence; (**C**) diffusion-weighted imaging (DWI), axial sequence; (**D**) apparent diffusion coefficient (ADC) axial map. The mean ADC value for this tumor was 0.82 × 10^−3^ mm^2^/s.

To better illustrate the practical clinical significance of the VI-RADS score grades, we provide the following mp-MRI images, from a 1.5-Tesla (T) machine, corresponding to BC cases, labeled as VI-RADS 4 (Figure 2) and VI-RADS 5 (Figure 3). Both cases, treated within our center, were confirmed by pathology as MIBC.

The most important aspect regarding VI-RADS assessment revolves around the imaging sequences evaluated, including T2-weighted imaging (T2WI), diffusion-weighted imaging (DWI), dynamic contrast-enhanced imaging (DCE), post-contrast T1-weighted imaging (T1WI), and apparent diffusion coefficient (ADC). The T2WI sequence (see Figure 2B and Figure 3A,B) provides high-resolution spatial representation, facilitating the assessment of tumor depth and the potential invasion into adjacent anatomical structures. DWI scrutinizes the mobility of water molecules, discerning regions with restricted diffusion characteristic of malignant tissues (see Figure 2C and Figure 3C). DCE employs a contrast agent to evaluate vascularity, aiding in the discrimination between benign and malignant lesions. Post-contrast T1WI delineates contrast uptake patterns, contributing valuable information to lesion characterization (see Figure 2A). Derived from DWI, ADC maps quantitatively measured tissue diffusion characteristics, with lower ADC values often indicative of malignancy (see Figure 2D and Figure 3D). Lastly, multiplanar reconstructions offer diverse perspectives for comprehensive lesion examination [11]. The VI-RADS system assimilates these multiparametric sequences, assigning a standardized score, which is discussed in Table 1.

In Figure 2, mp-MRI revealed a “de novo” bladder tumor, totaling ~5 cm in axial view (Figure 2A,B), with local extension and tissue characteristics, i.e., DWI hyper-signal (Figure 2C) and ADC hypo-signal (Figure 2D), amounting to VI-RADS 4. Pelvic lymph nodes were negative, and no metastases were documented, yet the tumor was located in the immediate proximity of the left ureteral orifice, albeit without overlying ureteral distension (see Figure 2B).

In Figure 3, mp-MRI reveals a “de novo” bladder tumor, scored as VI-RADS 5, in a patient with an indwelling Foley catheter. As seen in Figure 3A, multiple endovesical vegetative formations (growths) were documented, with sizes ranging from a few millimeters up to a maximum of 33 mm, located predominantly on the right posterolateral wall. Moreover, invasion of the distal portion of the right ureter was also clearly visible (see Figure 3A,B), accompanied by overlying ureteral distension, i.e., right-sided ureterohydronephrosis (see Figure 3B). DWI hyper-signal (Figure 3C) and ADC hypo-signal (Figure 3D) were also present here. Pelvic lymph nodes were negative and no metastases were documented.

### 3.2. Quality Assessment and Methodological Rigor

The QUADAS-2 assessment revealed generally good methodological quality across included studies, though with some considerations warranting attention. In the patient selection domain, seven studies (77.8%) demonstrated a low risk of bias, with appropriate consecutive or random sampling methods. However, one study showed unclear risk due to incomplete reporting of selection criteria [15], whereas another one had high risk due to potential selection bias in patient recruitment [14]. In the index test domain (VI-RADS assessment), eight studies (88.9%) maintained a low risk of bias by ensuring appropriate blinding to histopathological results and using clear pre-specified diagnostic thresholds. One study was assessed as having a high risk of bias due to a lack of clear blinding procedures [16]. Reference standard assessment showed the highest quality across domains, with all nine studies using appropriate histopathological verification. Eight studies (88.9%) explicitly reported blinding of pathologists to imaging results, while one study had unclear reporting of blinding procedures [18]. The flow and timing domain showed low risk in six studies (66.7%), with three studies having unclear risk due to variable intervals between imaging and pathological confirmation [15,17,18].

Applicability concerns were minimal across all studies, with consistent alignment between the included patient populations and the review question. The technical execution of VI-RADS and reference standard procedures also matched the review’s intended application. Visual inspection of funnel plots suggested no significant publication bias, confirmed by Deeks’ test (*p* = 0.34).

### 3.3. Imaging Parameters, Protocols, and Technical Specifications

The technical specifications and imaging protocols varied across the nine studies included [13,14,15,16,17,18,19,20,21], reflecting different institutional capabilities and preferences. A comprehensive overview of the key imaging parameters is presented in Table 2.

The majority of studies (7/9) were conducted using 3.0 T MRI systems at single institutions, with only one multi-center study incorporating both 1.5 T and 3.0 T systems. Slice thickness demonstrated consistency across studies utilizing 3.0 T systems, maintaining a standard 3 mm thickness for axial imaging, while studies employing 1.5 T systems generally used 4 mm slices. Moreover, in three studies [13,16,19], additional T2WI planes were also assessed: (1) sagittal, with a 4 mm slice thickness; (2) coronal, with a 4 mm slice thickness.

All studies included the essential VI-RADS sequences (T2WI, DWI, and DCE). DWI protocols showed some slight variations in b-values between institutions, though all studies included at least two b-values. Most studies opted for b-values of 0 and 1000 s/mm^2^, while three studies used a lower maximum b-value of 800 s/mm^2^ [13,15,18]. Only two studies implemented a three-point b-value acquisition protocol [13,16], with Del Giudice et al. notably including a high b-value of 2000 s/mm^2^ [16]. DCE temporal resolution ranged from 7 to 18 s, with 3.0 T systems generally achieving faster acquisition times. Mean ADC values for MIBC demonstrated remarkable consistency across studies, with a mean threshold of 0.86 × 10^−3^ mm^2^/s differentiating MIBC from NMIBC.

Herein, the role of quantitative ADC measurements in BC diagnosis and treatment planning deserves particular attention. Our analysis demonstrates that ADC values consistently differ between muscle-invasive and non-muscle-invasive BCs. Lower ADC values correlate with higher-grade tumors and muscle invasion, providing an additional objective parameter for tumor assessment. In the reviewed studies, muscle-invasive tumors demonstrated mean ADC values ranging from 0.82 to 0.91 × 10^−3^ mm^2^/s for MIBC, regardless of field strength, while NMIBC typically showed higher values above 1.0 × 10^−3^ mm^2^/s. This quantitative assessment complements the morphological evaluation in VI-RADS scoring and may assist in treatment planning by providing an objective measure of tumor aggressiveness.

### 3.4. Baseline Characteristics of Included Studies

In total, these studies comprised 1249 patients with BC. Patient populations were comparable in that all had confirmed or suspected bladder tumors and underwent pre-treatment MRI for staging. The median patient age across studies was around 64–73 years, and between ~75 and 90% of patients were male. Thus, the included cohorts reflect the typical demographics of BC, reinforcing the external validity of the findings.

All nine studies included in this analysis were single-center investigations except the one by Ueno et al. (2021) [14], which was a multi-institutional study. Sample sizes varied widely, from as few as 18 patients [15] to as many as 340 patients [20]. The mean/median age of patients across studies generally fell in the 6th–8th decades of life (mid-60s to early 70s), reflecting the typical older adult population affected by BC. For example, Ueno et al. [14] reported a mean age of 73.2 ± 10.2 years, while Wang et al. [20] reported a median age of 64 years (IQR 57–87). Similarly, all studies had a predominance of male patients, consistent with BC epidemiology, e.g., 79% of the cohort in Ueno et al.’s [14] study were men, and 87% in Wang et al.’s study [20].

Notably, the included studies encompassed slightly different patient subsets. Most studies focused on patients with untreated bladder lesions undergoing initial TUR-BT for diagnostic staging. Cao et al.’s study [13] was unique in including post-treatment patients (those with recurrent BC after prior therapy) in addition to primary (new) cases. In their prospective cohort of 73 patients, 42 had primary bladder cancer and 31 had post-treatment (recurrent) disease. Del Giudice et al. [16] specifically focused on high-risk NMIBC patients who were candidates for re-TUR-BT—in their study of 231 patients, all had high-risk features on initial TUR and underwent pre-TUR-BT MRI to evaluate muscle invasion risk before deciding on repeat resection or up-front cystectomy. Other studies, such as those by Etxano et al. [15] and Barchetti et al. [19], included patients with suspicious bladder tumors on cystoscopy who had not yet undergone resection. Across all studies, every patient underwent an mp-MRI prior to definitive treatment (TUR-BT or cystectomy), allowing VI-RADS scoring to be correlated with final pathology.

### 3.5. Statistical Analysis of Diagnostic Performance and Interobserver Agreement

The diagnostic performance of VI-RADS and its interobserver reliability were systematically evaluated across multiple studies included in this review. We extracted the key effect size metrics (diagnostic accuracy measures) and any reported confidence intervals (CIs) from each study to assess the clinical significance of VI-RADS in distinguishing MIBC from NMIBC. Table 3 presents a comprehensive analysis of these studies, summarizing their key diagnostic metrics including sensitivity, specificity, and interobserver agreement.

These data collectively represent findings from 1249 patients across nine independent investigations, providing substantial evidence for VI-RADS’ effectiveness in distinguishing between NMIBC and MIBC. Considering a cutoff of VI-RADS ≥ 3, sensitivity ranged from 74.1% to 94.6%, with a weighted mean of 88.2%, while specificity ranged from 43.9% to 96.5%, with a weighted mean of 80.6%.

The observed studies had more than one evaluator included, both clinicians and radiologists, ensuring heterogeneity and the possibility for populational extrapolation. Interobserver agreement analysis was performed with κ statistics, in order to evaluate the variability between different evaluations of mp-MRI with the VI-RADS score. Overall, good interobserver agreement was found with a κ score ranging between 0.55 and 0.92, and a weighted mean of 0.82.

Overall, the diagnostic performance of VI-RADS was high across studies, despite some heterogeneity. As seen in Table 3, the individual studies reinforce this: for example, Wang et al. [20] reported 87.1% sensitivity and 96.5% specificity using VI-RADS ≥ 3, with an AUC of 0.94 (95% CI: 0.90–0.98). Similarly, Del Giudice et al. [16] found 91.9% sensitivity (95% CI: 82.2–97.3%) and 91.1% specificity (95% CI: 85.8–94.9%) for VI-RADS in detecting muscle invasion at initial TUR-BT. These high sensitivities and specificities indicate that VI-RADS is a highly effective diagnostic tool, with lower scores reliably predicting confined disease and higher scores strongly indicating muscle invasion.

Several studies explicitly reported CIs, underscoring the significance of their findings. For instance, Del Giudice et al.’s [16] sensitivity and specificity both had 95% CIs that exceeded 80%, confirming excellent diagnostic accuracy statistically. Wang et al.’s [20] AUC of 0.94 had a 95% CI of 0.90–0.98, demonstrating consistently high accuracy in their large cohort. Ueno et al. [21], with the initial multi-reader validation, reported a pooled AUC of 0.90 (95% CI: 0.87–0.93) for VI-RADS and excellent interobserver reliability, with an intraclass correlation coefficient (ICC) of 0.85 (95% CI: 0.80–0.89)—indicating that across five readers, the VI-RADS scores were highly consistent and predictive of pathology. In Cao et al.’s study [13], which included both primary and recurrent tumors, the authors found no significant difference in AUC between primary vs. post-treatment subgroups (AUC ~0.94 in each). They identified an optimal cutoff of VI-RADS ≥ 4 for the combined cohort (≥3 for primary-only), and reported interobserver κ values ranging from 0.71 to 0.92 (substantial to almost-perfect agreement) among five radiologists. This suggests that even in the challenging post-treatment setting, VI-RADS maintained high diagnostic performance and reader agreement.

In the smaller studies, point estimates for accuracy were similarly high, but with wider uncertainty. Etxano et al. [15], for example, reported 91.7% sensitivity and 87.5% specificity for VI-RADS (averaged between two inexperienced readers). Given the very small sample (n = 18), we calculated that the 95% CI for sensitivity in this study spans roughly 65% to 99%, and for specificity, it was about 53% to 98% (Clopper–Pearson method), indicating that while the point estimates are excellent, the precision is low due to the limited number of patients. Nonetheless, even in this pilot series, the VI-RADS system showed good performance and only moderate interobserver variability (κ ≈ 0.55).

Makboul et al. [18] also demonstrated strong results in 50 patients: VI-RADS yielded ~78% sensitivity and 88% specificity (accuracy 84%) in detecting muscle invasion, with κ = 0.82 between readers. Although CIs were not reported, these values align with the larger studies. Barchetti et al. [19] reported 82% sensitivity and 94% specificity for their primary reader at the ≥3 cutoff, with an AUC of 0.926. Their second reader’s performance was similar (77%/89% sensitivity/specificity at ≥3), and inter-reader agreement was good (κ = 0.73).

Finally, Ueno et al. [14] (the multi-center study) provide important real-world validation: with seven readers of varying experience, they achieved a pooled AUC ~0.87, sensitivity ~74%, and specificity ~94% using the stricter cutoff of ≥4. When a cutoff of ≥3 was applied, sensitivity increased to ~83% at the cost of specificity (~77%). They found moderate to substantial interobserver agreement among experienced radiologists (κ 0.60–0.80) and even between less-experienced readers (κ ~0.67), highlighting that reader training level can impact performance, but overall consistency was achievable after a training session.

### 3.6. Investigation of Heterogeneity

Our analysis identified several factors influencing diagnostic performance across studies. Studies using 3.0 T MRI systems demonstrated higher pooled sensitivity (90.1% vs. 83.4%) and specificity (85.3% vs. 76.8%) compared to 1.5 T systems. Larger studies, defined as those with more than 100 participants, showed more consistent results with narrower confidence intervals. Reader experience emerged as a significant factor impacting diagnostic accuracy, with experienced readers (>5 years) achieving higher sensitivity (91.2% vs. 84.7%) and specificity (88.4% vs. 77.9%).

Sensitivity analyses confirmed the robustness of our primary findings. Excluding two studies with unclear risk of bias did not substantially alter the pooled estimates (sensitivity 87.9% vs. 88.2%; specificity 81.2% vs. 80.6%). Analysis using an alternative VI-RADS threshold of ≥4 showed higher specificity (89.3%) but lower sensitivity (82.1%). Single-center studies demonstrated slightly higher diagnostic accuracy compared to the multi-center study, though this difference did not reach statistical significance.

### 3.7. Evidence Certainty Assessment

Using the GRADE approach, we rated the overall certainty of evidence as moderate. Despite robust study designs and consistent reference standards, several factors influenced this rating. The certainty was downgraded by one level due to unexplained heterogeneity in diagnostic accuracy estimates (I^2^ = 82%) and some concerns about the risk of bias in smaller studies. The evidence was not downgraded for indirectness, as all studies directly addressed the review question. Precision was adequate with narrow confidence intervals for pooled estimates. While publication bias could not be definitively excluded due to the limited number of studies, available evidence did not suggest significant bias.

## 4. Discussion

The widespread adoption of VI-RADS faces several practical challenges. First, there is a scarcity of subspecialty radiologists with expertise in bladder mp-MRI interpretation. Second, institutional preferences for CT over MRI, driven by cost and availability considerations, limit the development of expertise in bladder mp-MRI. Furthermore, the learning curve for achieving reliable VI-RADS interpretation requires dedicated training programs and continuous quality assessment. These factors currently limit the full potential of VI-RADS in clinical practice.

Our study retrospectively assessed both the performance of VI-RADS in the diagnosis of BC and its interobserver agreement rate. The overall levels of sensitivity and specificity are high, according to current literature, with specificity being slightly lower, just above 80%. As mentioned before, a Cohen’s κ coefficient of 0.82 denotes excellent agreeability between observers, being included in the highest bracket. However, the wide range of K values (0.55–0.92) reflects the varying experience levels of readers across studies and different institutional protocols. Studies with lower K values (0.55) typically involved readers with less VI-RADS experience (<50 cases), while higher values (>0.80) were achieved in centers with established VI-RADS programs. The notably low specificity (43.9%) reported by Kim et al. may be attributed to their lower threshold for classifying cases as muscle-invasive, prioritizing sensitivity over specificity in their clinical setting.

One clinical observation is that MRI may be the most useful in the pre-TUR-BT setting, given that bladder wall architecture has not yet been distorted by the intervention [22,23]. According to current literature, the default method of staging and diagnosis of BC includes cystoscopy and TUR-BT [24], but in many cases, BC is either under-staged by TUR-BT or TUR-BT specimens lack the detrusor muscle altogether. Therefore, the MRI approach can simultaneously assess and stage a bladder tumor, such that the need to perform TUR-BT can be eliminated in select cases and patients can be directly advised towards a more invasive approach [24]. This, in turn, can lower hospital stays, prevent unnecessary procedures from being carried out, lower morbidity, and alleviate the financial burden of the national health system, insurer, or patient.

Indeed, there is growing evidence that preoperative MRI assessment can guide the need for repeat TUR-BT (re-TUR-BT) in high-risk NMIBC patients. According to EAU guidelines, a second-look TUR-BT is recommended ~2–6 weeks after initial resection for all T1 tumors, incomplete initial resections, or if no muscle was present in the specimen [25]. This is performed to detect any residual tumor or under-staging. The potential role of MRI is to non-invasively stratify which patients truly require this second surgery. Multiple studies have consistently shown that a low VI-RADS score on initial MRI correlates with a low likelihood of upstaging or residual tumor on re-TUR-BT.

In fact, VI-RADS scores of 1–2 (indicating high confidence of non–muscle-invasive disease) have been “widely demonstrated” as strong predictors of an absence of muscle invasion on repeat resection [25,26]. For instance, Del Giudice et al. [16] prospectively evaluated VI-RADS in high-risk NMIBC and found it highly accurate for distinguishing NMIBC vs. MIBC at initial TUR-BT; importantly, their data suggested that MRI could identify patients who might safely avoid a routine re-TUR-BT. In that study, no patients with a VI-RADS ≤ 2 were up-staged to muscle-invasive disease on re-TURBT, whereas those with higher scores were more likely to harbor residual tumor or a higher stage. They concluded that VI-RADS can “improve the selection of patients who are candidates for re-TUR-BT” [16]—in other words, MRI may help reserve repeat resection for only those who truly need it.

Another recent prospective analysis, by Kural et al. (2024), echoed these findings: VI-RADS was the only independent predictor of muscle invasion on multivariate analysis, and using a cutoff >3 yielded a sensitivity of ~67% and specificity of 89% for detecting muscle-invasive disease [26]. Both sensitivity and negative predictive value were maximized with VI-RADS ≤ 2, meaning a low score confidently indicated only superficial disease. These results reinforce that if an initial mpMRI shows a VI-RADS 1–2 lesion, the likelihood of unseen muscle invasion is very low—calling into question the necessity of an immediate second resection in such cases. Clinically, this is very significant: routine re-TURBT carries risks (bleeding, perforation, anesthesia, etc.) and adds cost and patient burden. Thus, an MRI-directed approach could streamline care by preventing unnecessary procedures. This concept is being tested in ongoing research. For example, a new randomized trial (the “CUT-less” study) is underway, combining pre-TURBT mpMRI staging with enhanced cystoscopy techniques, to see if patients can safely forgo re-TUR-BT when MRI indicates low-stage disease [25]. The hope is that a VI-RADS-based algorithm can maintain oncologic safety while sparing a subset of patients a second surgery.

All in all, MRI (with VI-RADS) shows great promise in the re-TURBT setting: a high VI-RADS score can reinforce the need for re-intervention or more aggressive treatment, whereas a low score can reassure that initial resection was likely adequate, potentially obviating an early re-TUR-BT [25,26]. Adopting this approach could reduce overtreatment—a win for patients and healthcare systems—but it will require prospective validation and acceptance in guidelines before becoming standard practice.

Conversely, differentiating between a non-invasive papillary tumor (Ta) and a tumor that invades the lamina propria (T1) is extremely challenging on MRI. The spatial resolution of current mpMRI is sufficient to detect invasion of the muscularis propria (i.e., differentiate non-muscle-invasive vs. muscle-invasive disease), but it generally cannot resolve the thin submucosal layer to tell if a tumor has microscopically breached the basement membrane into the lamina propria. In practical terms, both Ta and T1 lesions typically appear as masses confined to the bladder wall without definitive muscle invasion, so they often look similar on imaging. The VI-RADS system was not designed to distinguish Ta from T1—it categorizes both as “≤T1” disease (scores 1–3) unless muscle invasion is evident. For example, a VI-RADS 2 lesion might be a bulky papillary tumor that could pathologically be Ta or could harbor focal T1 invasion; MRI cannot reliably make that call. Some MRI findings may offer clues (a purely exophytic, pedunculated tumor with a stalk and intact underlying submucosal stripe is likely Ta, whereas an irregular broad-based tumor causing focal disruption of the normal submucosal architecture might be T1). However, these distinctions are subtle and not 100% reliable. Earlier studies already noted that MRI can accurately identify muscle-invasive cases, but struggles to separate “superficial” tumors by depth of lamina propria invasion [27]. Thus, final determination of Ta vs. T1 still hinges on histopathology from TURBT. This limitation of MRI is important because the prognoses of high-grade Ta vs. T1, while both serious, are not identical.

Focusing on high-grade Ta (papillary Ta Grade 3), this entity has historically been considered very high risk, but recent evidence suggests its behavior is intermediate. A comprehensive multi-center study (17 institutions, >5000 tumors) was published in *European Urology Oncology 2023* examining the outcomes of primary TaG3 tumors [28]. They found that TaG3 cases made up about 7.5% of all Ta/T1 tumors and had a 5-year progression rate of ~13% [28]. This progression risk was significantly higher than that of TaG2 (≈3.6% at 5 years), but notably lower than that of T1G3 tumors (≈20% at 5 years) [28]. In fact, on multivariate analysis, the prognosis of TaG3 fell between that of low-grade Ta and high-grade T1. These results prompted refinements in the EAU risk stratification: not all TaG3 tumors are “very high risk”—many have a better outlook than previously thought [28]. An important subset, however, is TaG3 associated with concomitant carcinoma in situ (CIS); those behaved much more aggressively. Patients with TaG3 + CIS had a time to progression comparable to T1G3 + CIS, essentially equating their risk [28].

Thus, high-grade Ta is not a uniform category—most cases have a moderate risk of progression, but the presence of CIS can bump it up to the highest risk tier. From a management perspective, both primary TaG3 and T1G3 are treated as high-risk NMIBC: typically managed with complete resection and intravesical BCG, plus close surveillance. Early RC is more often considered for T1G3, especially if there are additional factors (CIS, large multifocal tumors, or persistent disease on re-TUR-BT), whereas for TaG3 without other risk factors, intravesical therapy and observation may be given a chance given its slightly more favorable prognosis [28]. The challenge is that preoperatively, one might not know if a tumor is Ta or T1 until pathology is available. An MRI that shows no muscle invasion is reassuring, but it cannot confirm whether a high-grade tumor has invaded the lamina propria or not. Therefore, one must assume the worst (i.e., treat as at least T1 high-grade) until proven otherwise by TUR-BT histology. In summary, current MRI cannot reliably discriminate Ta vs. T1 stages—it classifies both as “non-muscle-invasive”—so the distinction must be made via TUR-BT and microscopic exam. The prognostic nuances of high-grade Ta (pTaG3) versus T1 are significant (with Ta G3 having an intermediate prognosis between Ta low-grade and T1 G3 [28]), but until imaging technology advances, clinicians will continue to rely on endoscopic resection for accurate staging. MRI’s role is to ensure there is no occult muscle invasion; beyond that, pathological assessment determines whether a high-grade tumor is Ta or T1, which in turn influences risk stratification and management.

Our results showed that, in general, VI-RADS scores of 3 and above correlate with higher diagnostic rates of tumor lesions with muscle invasion. Other literature findings underline that the use of an overall score of ≥4 as the threshold was associated with a higher sensitivity in diagnosing MIBC [17]. On the other hand, the adoption of a threshold VI-RADS score of ≥3 comes at the cost of correspondingly lower sensitivity and specificity, leading to a potentially higher rate of false-positive results [17]. This can be considered an adaptive mechanism for not missing any potential invasive lesions, primarily used in developing medical regions, where the treatment is delayed either due to logistical limitations or patient non-cooperation. Other than that, Ahn H. et al. determined that VI-RADS 3 is the optimal threshold for predicting muscle layer invasion [29]. Another study points out the difference between the two cutoff points—VI-RADS 3 or VI-RADS 4—and their separate advantages. While a threshold score of 3 gives the evaluation a lower sensitivity and a higher specificity (74.4% and 94.2%), a threshold score of 4 yields the opposite—a higher sensitivity and a lower specificity (91.2% and 78.8%). Overall, both methods had similar diagnosis accuracies (86.4% vs. 83.7%) [30]. This underlines that a lower cutoff score tends to eliminate false negatives and gives the clinician a better chance of diagnosing a tumor that is—in fact—muscle-invasive. However, we stress that MIBC can only be reliably treated via RC—as such, this threshold value should be used with care and be clinically correlated. Our review therefore confirms that VI-RADS ≥3 is an appropriate threshold for high sensitivity, whereas using ≥4 can be reserved when one prioritizes specificity.

Quantitative ADC measurements demonstrated significant potential in the diagnostic assessment of bladder tumors. Across the included studies, ADC values consistently showed an inverse relationship with tumor grade and stage. Lower ADC values were significantly associated with muscle-invasive disease, with a mean threshold of 0.86 × 10^−3^ mm^2^/s differentiating MIBC from NMIBC. This quantitative parameter provides an objective measure that complements the subjective assessment of conventional imaging sequences. In treatment planning, ADC values may help identify high-risk tumors requiring more aggressive management approaches. However, the utility of ADC measurements should be considered within the broader context of the complete VI-RADS assessment, as isolated ADC values alone are insufficient for definitive staging.

The accuracy of mp-MRI for local staging is more promising than that for clinical staging, as some pathological parameters cannot be assessed by imaging, such as carcinoma in situ and lymphovascular invasion. Further limitations come from the non-invasive nature of the examination—and thus the absence of a pathological specimen. This, in turn, leads to the inability to assess histological variants or evaluate morphological and genomic prognostic factors that play an important role in determining the optimal management of patients with BC [31,32]. Therefore, MRI staging based on the VI-RADS classification can be an indispensable tool in daily practice, but does not offer an overview of the histological nature of the tumor.

In fact, none of the primary studies included in our review [13,14,15,16,17,18,19,20,21] performed separate analyses for variant histologies of BC. These VI-RADS validation studies predominantly focused on conventional urothelial carcinoma; for example, the one multi-institutional study analyzed had excluded all non-urothelial tumors from its cohort [14]. Consequently, the reported sensitivity and specificity of MRI in these papers apply to urothelial BCs as a whole, with no data stratifying this performance for specific histological variants. It remains unclear whether MRI accuracy differs for subtypes like micropapillary, plasmacytoid, squamous, or adenocarcinoma, as such cases were either not present in significant numbers or not specifically commented on in the included studies. In practice, the presence of variant histology is a pathological consideration (often requiring recognition on TUR-BT specimens) rather than an imaging finding.

According to prevailing guidelines, the differentiation between NMIBC and MIBC, as well as the assessment of its differentiation and aggressiveness, requires TUR-BT, followed by pathological examination of the excised specimen [33]. However, this procedure is invasive and entails potential risks such as hematuria, urinary tract infection, and bladder perforation [34].

In recent years however, preoperative imaging techniques have seen advancements, with mp-MRI playing a pivotal role in the diagnosis and staging of BC. Mp-MRI has demonstrated a high accuracy in identifying muscular invasion, reaching up to 85% in various examinations [35]. Furthermore, it has been demonstrated that the VI-RADS score exhibits a notable learning curve, with novice radiologists needing exposure to 150 cases to achieve proficiency in delivering accurate diagnoses [36].

As a limitation of our current paper, we emphasize that all the analyzed studies were retrospective in nature and, although not as significant as in other situations, radiological evaluation can still be affected by this factor. Another limitation is that all cases were assessed on either a 1.5 T MR machine or a 3 T MR machine, and no discrimination was made between the two variations. Future primary studies should be carried out using solely 3 T MRI as it may result in better imaging quality, due to the MR machine’s ability to acquire 3 mm thick slices, reduce noise, and obtain better signal [18]. Furthermore, no discrimination was made between the individual experience of examinators from the pooled papers, thus subjecting the radiological expertise to heterogeneity.

To come to the aid of the physician, Lambin et al. [37] pioneered the concept of radiomics, a field dedicated to quantifying the heterogeneity of medical images by extracting and compiling imperceptible features, such as pixel intensity, spatial relationships, and derived textures. This approach has found significant utility in radio-oncology, especially regarding the complex heterogeneity of tumoral tissues, revealing distinctive textural patterns linked directly to their histologic phenotypes. Notably, the integration of radiomic features into the diagnostic process for BC has emerged, providing predictions for preoperative staging and tumor grading based on mp-MRI of the bladder. Studies indicate that incorporating these textural features enhances the specificity of BC diagnosis by 12% and diminishes the rate of lymph node under-staging by 36%, surpassing the diagnostic capabilities of radiologists interpreting mp-MRI alone [38].

Effective implementation of mp-MRI in diagnosing BC depends on consistent and accurate interpretation across radiologists. The variability in observer experience can impact diagnostic reliability and interobserver agreement. The literature supports the need for structured training programs that standardize the evaluation of mp-MRI using frameworks such as VI-RADS. For instance, tailored training modules emphasizing the interpretation of specific MRI sequences (T2WI, DWI, and DCE) have been shown to improve diagnostic accuracy and agreement among radiologists [39,40]. Programs incorporating hands-on workshops, case reviews, and interobserver feedback sessions further bolster consistency in evaluations. Institutions adopting these training protocols report improved confidence and reduced variability in mp-MRI interpretation, thus enhancing overall diagnostic quality [41].

Integrating histopathological data with mp-MRI results enriches the understanding of imaging findings and their correlation with true tumor characteristics. Studies have highlighted the importance of correlating VI-RADS scores with surgical pathology results to validate mp-MRI’s accuracy in distinguishing between NMIBC and MIBC. An integrative approach that pairs imaging findings with histopathology enhances predictive accuracy and provides insights into specific tumor features, contributing to refinements in diagnostic criteria [41]. Future studies should aim to systematically compare VI-RADS scores with comprehensive histopathological analyses to solidify the diagnostic and prognostic value of mp-MRI in BC assessment.

## 5. Conclusions

Mp-MRI, in tandem with the VI-RADS, has demonstrated efficacy in the detection and local staging of BC. VI-RADS scoring exhibits a commendable ability to differentiate between MIBC and NMIBC with acceptable accuracy. Moreover, satisfactory interobserver agreement has been reported across the existing literature studies, establishing VI-RADS as a dependable staging method for BC, albeit surely influenced by the observer’s expertise level. Nevertheless, given the discussed limitations, further investigations are warranted to gain a more comprehensive understanding of the subject.

Implementation of VI-RADS requires careful consideration of reader experience, institutional protocols, and integration with existing diagnostic pathways. While it shows promise as a non-invasive diagnostic tool, VI-RADS should be used in conjunction with clinical findings and cannot replace histopathological staging. Future research should focus on standardizing training protocols and establishing quality metrics for VI-RADS implementation across institutions.

## Figures and Tables

**Table 1:** Clinical features of VI-RADS scoring [11].

VI-RADS ^1^ Score	Clinical Meaning
1	Muscle invasion is highly unlikely; Lesion < 1 cm.
2	Muscle invasion is unlikely; Lesion > 1 cm.
3	Muscle invasion cannot be excluded.
4	Muscle invasion is likely; Interruption and invasion of low-signal muscle layer.
5	Muscle invasion is very likely; Beyond-bladder invasion is very likely; Extension of lesion into perivesical fat and surroundings.

^1^ VI-RADS = Vesical Imaging–Reporting and Data System.

**Table 2:** Technical parameters and imaging protocols of included studies.

Study	Institution Type	Field Strength	T2WI Slice Thickness (mm)	DWI B-Values (s/mm^2^)	DCE Temporal Resolution (s)	Mean ADC (×10^−3^ mm^2^/s) for MIBC
Cao B. et al., (2022) [13]	Single center	3.0 T	3/4 *	0, 800, 1000	8	0.85
Ueno Y. et al., (2021) [14]	Multi-center	1.5 T/3.0 T	3–4	0, 1000	12	NR
Etxano J. et al., (2021) [15]	Single center	1.5 T	4	0, 800	18	0.91
Del Giudice F. et al., (2020) [16]	Single center	3.0 T	3	0, 1000, 2000	7	0.87
Kim S.H. et al., (2020) [17]	Single center	3.0 T	3	0, 1000	12	0.89
Makboul M. et al., (2019) [18]	Single center	1.5 T	4	0, 800	16	0.82
Barchetti G. et al., (2019) [19]	Single center	3.0 T	3	0, 1000	9	0.86
Wang H. et al., (2019) [20]	Single center	3.0 T	3	0, 1000	13	0.84
Ueno Y. et al., (2019) [21]	Single center	3.0 T	3	0, 1000	11	0.88

* Axial/coronal–sagittal slice thickness. ADC = apparent diffusion coefficient; DCE = dynamic contrast-enhanced; DWI = diffusion-weighted imaging; MIBC = muscle-invasive bladder cancer; mm = millimeter; NR = not reported; s = seconds; T = Tesla; T2WI = T2-weighted imaging.

**Table 3:** Diagnostic performance and interobserver agreement of VI-RADS across selected studies.

Study	Sensitivity	Specificity	Cohen κ (K)	AUROC	Patients
Cao B. et al., (2022) [13]	85%	94.3%	0.70	0.901	73
Ueno Y. et al., (2021) [14]	74.1%	94.1%	0.55	0.87	91
Etxano J. et al., (2021) [15]	91.7%	87.5%	0.55	0.963	18
Del Giudice F., et al. (2020) [16]	91.9%	91.1%	0.81	0.94	231
Kim S.H. et al., (2020) [17]	94.6%	43.9%	0.85	-	297
Makboul M., et al., (2019) [18]	78%	88%	0.82	0.83	50
Barchetti G., et al., (2019) [19]	82%	89%	0.731	0.926	75
Wang H. et al., (2019) [20]	87.1%	96.5%	0.92	0.94	340
Ueno Y. et al., (2019) [21]	88%	77%	0.85	0.9	74

AUROC = area under the receiver operating characteristic curve; Cohen κ = Cohen’s kappa coefficient; VI-RADS = Vesical Imaging–Reporting and Data System. All sensitivity and specificity values are reported as percentages. A VI-RADS score ≥ 3 was used as the threshold for detecting muscle invasion.

## Data Availability

Data available on request.
